# Measuring geographical disparities in waiting times for community-based specialist care - a novel statistical application

**DOI:** 10.1186/s13584-025-00702-7

**Published:** 2025-07-14

**Authors:** Havi Murad, Vicki Myers, Arnona Ziv, Rachel Wilf-Miron, Osnat Luxenburg

**Affiliations:** 1 Biostatistics & Biomathematics Unit, Data & Analytics Division, Sheba Medical Center, Ramat Gan, Israel; 2 Gertner Institute of Epidemiology & Health Policy Research, Sheba Medical Center, Ramat Gan, Israel; 3 School of Public Health, Faculty of Health & Medical Sciences, Tel Aviv University, Tel Aviv, Israel; 4 Data & Analytics Division, Sheba Medical Center, Ramat Gan, Israel; 5 Ministry of Health, Israel

**Keywords:** Waiting times, Piecewise linear quantile regression, Geographical disparities

## Abstract

**Background:**

Gaps in provision of healthcare by geographical location have been demonstrated around the world. To detect disparities in waiting times (WT), methods are required for accurate comparison. This study sought to demonstrate a novel statistical application to assess geographical differences in WT and time trends for community dermatologists in Israel between 2019 and 2023.

**Methods:**

WT were measured using available appointments and actual visits for all dermatologists in all four Israeli Health Maintenance Organizations (HMO). Piecewise quantile regressions were conducted for two periods: 2019–2021 and 2022–2023. Additive and multiplicative models estimated absolute and relative differences in median WT between regions. Expected WT, time trends, and differences from the national level were calculated. Validation of the statistical model was conducted on 2024 data.

**Results:**

Between 2019 and 2021, median national WT began at 17 days (95% CI 14–20), increasing annually by 2.5 days (+ 11%) until end of 2021. WT was shortest in Jerusalem (10 days) and longest in Tel Aviv (21 days). Increasing time trends were demonstrated in all regions except North and Haifa. The model successfully predicted 2024 national WT. By 2022–2023, national median WT began at 24.5 days (95% CI 21–28) and reached an annual increase of 8.5 days (+ 26%). WT was shortest in the North (14 days) and longest in Tel Aviv (40) and J&S (42 days). Increasing time trends were demonstrated in all regions except North and Haifa, with the greatest increase in Jerusalem and Centre regions (+ 12 days/year).

**Conclusions:**

WT for a dermatologist in the public healthcare system increased significantly between 2019 and 2023, particularly after the peak of the COVID pandemic, with increased disparities among regions. Advanced statistical modelling, such as piecewise quantile regression, highlights areas in need of intervention and can contribute to resource planning to help improve system efficiency. Future research should examine explanatory factors including physician-hours per population, patient scheduling preferences, as well as advancing tools to help direct patients to the most appropriate service to reduce burden on specialists.

**Supplementary Information:**

The online version contains supplementary material available at 10.1186/s13584-025-00702-7.

## Background

Waiting times (WT) are a key marker of quality and system performance in healthcare systems and are a key component of patient satisfaction. Long waiting times may affect health outcomes as well as perceived quality of care and may increase anxiety [1, 2]. Furthermore, long WT in the public health system results in greater use of private consultations, thus increasing disparities. Waiting times have been further exacerbated in recent years, affected by the COVID-19 pandemic (for example in UK and Canada [3, 4].

WT can be assessed by different methods, including the utilization of administrative data, patient surveys or secret shopper surveys. In a national survey conducted in Israel in 2018, approximately a third of patients were satisfied with WT for specialists; while a third of patients reported waiting over a month for a specialist appointment [5].

A novel algorithm was developed by the Israel Ministry of Health to routinely assess WT for community specialists, based on data from computerized appointment scheduling systems of all four Israeli Health Maintenance Organizations (HMOs). The algorithm uses available appointments for each community-based specialist practice and actual number of visits [6]. WT offered to HMO members for non-urgent care were calculated for two scenarios: “specific” or named physician and “any” physician in the region. The distribution of offered WT was calculated separately for each specialty and geographical region, combined to create the nationwide distribution. The leading statistical measure in the public reporting of the WT was the median.

Computed WT has been reported to policy makers and the public on a quarterly basis. Results showed that among the five most common specialties (orthopedics, ophthalmology, gynecology, otolaryngology, dermatology), the longest WTs at the national level in 2019 were for dermatology. For example median WT for a specific named physician in the second quarter of 2019 ranged from 11 days in ophthalmology to 20 days in dermatology; for any physician in a given area, median WT ranged from 6 days in gynecology to 13 days in dermatology [6]. Dermatology was therefore chosen as the first specialty to examine geographical disparities.

Gaps in provision of healthcare by geographical location have been demonstrated in Israel and around the world. Israel is divided into seven geographical areas according to location: South, Judea and Samaria (J&S), Jerusalem, Center, Tel-Aviv, North, and Haifa. The share of physicians in the center of Israel and in large cities is generally higher than their rate in the geographic periphery. For example, the number of physicians per population in the central Tel Aviv district in 2018–2020 was 2.3 and 1.9 times higher than their rate in the Northern and Southern districts, respectively [7]. Differences in waiting time by geographic area are unjust and can ultimately influence health outcomes.

In order to detect disparities in waiting times, methods are required for accurate comparison to assess whether apparent differences are significant. Firstly it is important to relate to both relative and absolute differences [8]. By examining the relative difference, we usually use ratios and can state that WT in a specific region is for example 3 times longer or 50% longer than in another region; while the absolute difference would tell us that WT in a specific region is for example 10 days longer than in another region.

Different methods have been used to compare waiting times, from simple statistical comparison (parametric or non-parametric) of average or median waiting times between specialties, regions or periods [9–11], to multivariate regression to compare waiting times over years [12]. In this paper we propose a new statistical application to compare waiting time for specialists over a period of five years (20 quarters) in the Israeli public health system, using a piecewise quantile regression for two periods (2019–2021 and 2022–2023, see Appendix A). Using a model instead of simple tests enables adjusting for important confounders such as seasonality and allows us to examine gaps in trends over time. Quantile regression, which is not frequently used for comparing waiting times, is more appropriate for such data due to their asymmetric heavy tailed distributions. The piecewise model [13, 14] allows to fit different slopes (trends over time) in the two periods mentioned above.

### Aim

This study aimed to demonstrate a novel statistical application to assess differences in waiting time (absolute and relative) and time trends for community specialists by geographic region over a period of 5 years from 2019 to 2023, including the period of the COVID pandemic and the aftermath, focusing on dermatology as a case study.

## Methods

### Data description

In Israel, WTs for community-based specialist care have been documented and publicly reported on a quarterly basis, since 2019. A prospective methodology allows for the calculation of WT for two scenarios: a specific (chosen or named) physician; and any physician in the region [6]. We chose to analyze specific over any physician, since in a survey among a representative sample of the adult Israeli population, of those who tried to make an appointment with a community-based specialist, 53% preferred a specific, named physician [15]. Furthermore, treatment by the same, known physician upholds the principle of continuity of care. Dermatology was chosen as one of the most common specialties, constituting 13% of all community-based visits to specialty care in 2024 [16] as well as due to long waiting times for an appointment [16]. Median time, rather than mean, was chosen as the leading statistic for both public reporting and dialogue with policy makers, since is neutralizes the effect of the “long tail” of very long WT among, for example, the most popular physicians.

WT for a ‘specific’ dermatologist was measured using available appointments offered for each specialist practice, according to their appointment schedule, taking into account the demand for specialist appointments by utilizing the actual number of visits in each practice during that month. For each consecutive day during the relevant period, the first 50 available appointments were extracted from the computerized scheduling system of each clinic. In parallel, the number of actual patient visits was collected from each physician’s practice during the same time period [6]. To measure WT for a “specific” (named, or chosen) physician, the daily demand is estimated by dividing the total number of actual visits to the physician by the number of days in the period (e.g. 31 days in March). Assuming a steady state (where demand is equal to supply), the number of first available appointments that should be used to calculate WT from the physician’s schedule each day (supply), is equal to the daily demand. All appointment types (frontal and remote) were included in the WT calculation.

Under the National Health Insurance law, all citizens are entitled to healthcare and are insured under one of four Health Maintenance Organizations (HMO). Data were collected from the 4 HMOs and combined to a national figure. Data were analyzed by the seven geographic regions. While there are smaller geographic units, such as districts and localities, region was chosen as a commonly used division, providing sufficient information of central vs. peripheral communities, and aiding ease of comparison and presentation.

### Data analysis

We propose a new statistical approach to characterize regional differences in median waiting times across time-periods (quarters), focusing on two different phases: (*i*) 2019–2021 (including the height of the Covid-19 pandemic); (*ii*) 2022–2023 (after the peak of the pandemic). Traditional regression, such as linear regression, models the mean of the dependent variable. However, we used quantile regression (Quantreg Procedure in SAS [15]), which models the relationship at different quantiles (e.g., median (50th percentile), 10th percentile, 90th percentile). This is particularly useful when data is skewed, as in the case of waiting times. To capture potential shifts in the relationship over time, we applied piecewise quantile regression, which fits multiple segments with different slopes, instead of a single straight line. These segments are separated by “knots” (breakpoints), where the relationship between variables changes. In our case, we defined two segments corresponding to the two time periods mentioned above, with the 13th quarter (1st quarter of 2022) as the knot, marking the transition between periods [13, 14] (see Appendix A). The model first evaluates whether a piecewise approach better fits the data by incorporating an interaction term (see Appendix A). It then estimates median waiting time at the start of each period and the trend (slope) over time for each region. Both additive and multiplicative models for absolute and relative differences in WT between regions were run, where the outcomes were median of waiting times (quantile level of 0.5) and log1-transformed median of WT (log1(x) = log(X + 1)) respectively. The log1 transformation was chosen in order to include WTs of zero. The independent variables were: region (South, Haifa, Jerusalem, Center, North, Tel-Aviv and J&S versus National (i.e., average of all 7 regions) as the reference category, Period (1,… 20 quartiles) as a continuous variable, indicator for restrictions during Covid-19 (Covid lockdowns were imposed nationally in 2nd quarter of 2020 and 1st quarter of 2021), indicator for Iron swords war (4th quarter of 2023), serial quarter in each year (1,2,3,4 where 1 was the reference category) to allow analysis of seasonality, interaction between period and region, and interaction for piecewise model testing at national level and for each region vs. national level.

Utilizing the additive piecewise quantile regression model, expected median WTs and their differences from the national median waiting time were calculated for the first quarter of 2019 and for the first quarter of 2022 for each region. In addition, the yearly slope (expected change in median WT per year) was calculated for each region in each of the two periods, as well as its difference from the ‘National’ slope. Similarly, utilizing the multiplicative model, expected median WTs, and their ratios in relation to the median waiting time at the national level were calculated for the first quarter of 2019 and for the first quarter of 2022 for each region. In addition, the slope (expected ratio in median WT per year) was calculated for each region and for national WT in each of the two periods.

To take into account multiple comparisons, a more stringent significance level of 0.007 (chosen for seven comparisons of each region with the national level) was used, according to Bonferroni [17].

For sensitivity analyses these models were also applied for other quantile levels of WT (0.5–0.9). These analyses will help to identify more general time trends.

Validation of our model against actual wait time data for the available first three quarters of 2024 was applied. Predicted median WT was calculated from our model and was compared to actual median WT using Mean Average Error (MAE) at the national and regional levels. When generating predictions for the first quarter of 2024, we took into account the effect of the war, as was done in the last quarter of 2023.

## Results

**Fig. 1:**
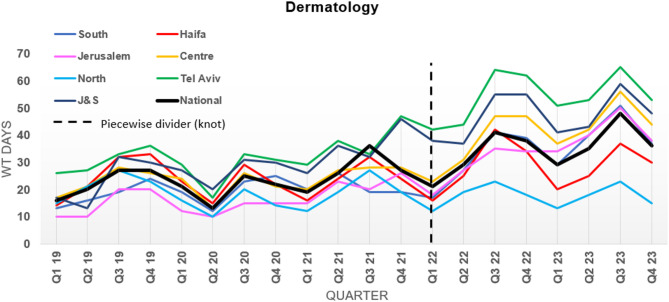
Median WT (days) for a specific (named) dermatologist by region 2019–2023 (Observed data)

Figure 1 presents crude observed median WT for a specific dermatologist by region as well as the national median WT. In the first period, 2019–2021, Tel Aviv region, (including the large central city of Tel Aviv), had the highest median WT throughout most of the study period, with J&S a close second. The lowest WTs were seen for Jerusalem and the Northern (peripheral) region. Large decreases in WT can be seen during the COVID lockdowns in the second quarter of 2020 and the first quarter of 2021, across all regions, and at the national level, alongside seasonal variation. In the second period, 2022–2023, Tel Aviv and J&S maintained the longest WT, followed by the Centre region in third place. WT in these three regions were much higher in the second compared to the first period. WT remained shortest in the Northern periphery. WT dropped for all regions in the 4th quarter of 2023 when Iron Swords War broke out.

### Model results and time trends

The results of the additive piecewise quantile regression model are presented in Tables 1 and 2. In the first period, at the national level, across all regions, expected waiting time in the 1st quarter of 2019 was 17 days (95%CI 14–20). The region of Jerusalem performed better with the shortest expected median waiting time of 10 days (95%CI 7–13; *P* < 0.0001 for difference from ‘National’). The Tel-Aviv region performed worse than the national average, with the longest expected median waiting time of 21 days (95%CI 16–26), however this was not significantly different from the national median (*P* = 0.14). All other regions were not statistically different from the national baseline median waiting times. With respect to the slope (time trend), the overall national median waiting time was expected to increase by 2.5 days per year (95%CI = 1–4) between 2019 and 2021. The North region performed better than the national level, yielding no statistically significant trends over time (-0.83, 95% CI- 2.37–0.71), with WT neither increasing nor decreasing. The expected annual increase was significantly worse in the J&S region with an expected median WT increase of 8 days per year (95% CI 6–10, *P* < 0.0001 for difference of slope from ‘National’); and in Tel-Aviv region with an expected increase in median WT of 7 days per year (95% CI 4–10; *P* = 0.002 for difference of slope from ‘National’). All other regions did not differ statistically from the national average with respect to the slope (trends over time).

In the second period, at the national level, expected waiting time for the 1st quarter of 2022 was 24.5 days (95%CI 21–28) and increased by 8.5 days annually (see slope in Table 2), i.e. 26% per year (see Table 4). The North region performed better with an expected median waiting time of 14 days (95%CI 11–18) and the Tel-Aviv and J&S regions performed worse than the national level, yielding expected median waiting times of 42 and 40 days respectively. All other regions were not statistically different from the national baseline median waiting times. WTs increased over time for all regions except for the North and Haifa regions, as in the first period. However, only the Jerusalem region performed borderline worse than the national level, yielding a 12-day annual increase in median WT (95%CI = 9–16, *P* = 0.07 for difference of slope from national), i.e. 39% increase per year (see Table 4).

**Table 1:** Additive piecewise quantile regression model results for WT by region, presenting absolute difference in 2019 and linear trend over time (slope); 2019–2021

Region	Baseline median WT (days) (95% CI)	Absolute Difference region vs. national WT	*p*	Slope* (time trend) (95% CI)	*P*	Difference in slope region vs. national (95% CI)	*P*
National	17.00 (14.13–19.87)			**2.50 (1.12–3.88)**	**0.0004**		
South	13.80 (10.56–17.04)	-3.20 (-6.68-0.28)	**0.07**	**3.30 (1.54–5.06)**	**0.0002**	0.80 (-1.10-2.70)	0.41
Haifa	18.50 (15.37–25.23)	1.50 (-3.25-6.25)	0.54	1.83 (-0.51-4.18)	0.126	-0.67 (-3.22-1.88)	0.61
Jerusalem	**9.70** (6.69–12.72)	**-7.30 (-10.82-(-3.78))**	**< 0.0001**	**3.97 (2.42–5.51)**	**< 0.0001**	1.47 (-0.50-3.43)	0.14
Centre	16.75 (12.68–20.82)	-0.25 (-4.61-4.11)	0.91	**3.50 (1.30–5.70)**	**0.002**	1.00 (-1.36-3.36)	0.41
North	16.83 (13.59–20.07)	-0.17 (-3.99-3.66)	0.93	-0.83 (-2.37-0.71)	0.289	**-3.33 (-5.40-(-1.27)**	**0.002**
Tel Aviv	20.86 (15.91–25.81)	3.86 (-1.22-8.93)	0.14	**7.07 (4.37–9.77)**	**< 0.0001**	4.57 (1.72–7.42)	**0.002**
J&S	16.17 (12.76–19.58)	-0.83 (-4.76-3.10)	0.68	**7.83 (6.07–9.60)**	**< 0.0001**	**5.33 (3.19–7.48)**	**< 0.0001**

**Bold** indicates significant at the level < 0.007

* indicates expected ratio for median number of days WT per year increase

WT, waiting time; CI, confidence interval, J&S, Judea and Samaria region

**Table 2:** **Additive piecewise quantile regression model results for WT by region**,** presenting absolute difference in 2022 and linear trend over time (slope); 2022–2023**

Region	Baseline median WT (days) (95% CI)	Absolute Difference region vs. national WT	*p*	Slope* (time trend) (95% CI)	*P*	Difference in slope region vs. national (95% CI)	*P*
National	24.50 (21.32–27.68)			**8.50 (5.34–11.66)**	**< 0.0001**		
South	23.70 (19.74–27.66)	-0.80 (-4.32- (2.72))	0.66	**10.67 (7.67–13.67)**	**< 0.0001**	2.17 (-1.38-5.72)	0.23
Haifa	24.00 (19.65–28.35)	-0.50 (-4.65-3.65)	0.81	2.50 (-1.79-6.79)	0.25	-6.00 (-10.86- (-1.14))	0.016
Jerusalem	21.60 (17.83–25.37)	-2.90 (-6.59- (0.79))	0.79	**12.10 (8.56–15.64)**	**< 0.0001**	3.60 (-0.34-7.54)	**0.07**
Centre	27.25 (22.76–31.74)	2.75 (-1.43-6.93)	0.20	**12.33 (7.52–17.14)**	**< 0.0001**	3.82 (-1.01-8.67)	0.12
North	**14.33** (11.04–17.62)	-10.17 (-13.64-(-6.70))	**< 0.0001**	0.94 (-1.80-3.69)	0.50	-7.56 (-11.01-(-4.10)	**< 0.0001**
Tel Aviv	**42.07** (37.00-47.15)	17.57 (12.84–22.30)	**< 0.0001**	**9.32 (5.01–13.63)**	**< 0.0001**	0.82 (-4.05-5.69)	0.74
J&S	**39.67** (35.88–43.46)	15.17 (11.36–18.98)	**< 0.0001**	**4.98 (1.48–8.48)**	**0.005**	-3.52 (-7.71-0.66)	0.1

Similar results, from the multiplicative quantile regression model, are presented in Tables 3 and 4.

At the national level, expected waiting time for the 1st quarter of 2019 was 18 days (95%CI 16–20), increasing by 11% each year until the end of 2021(see slope in Table 3). Again, the Jerusalem and South regions performed better with an expected median waiting time of 11 and 15 days respectively. Tel-Aviv region performed worse, yielding an expected median waiting times of 23 days, significantly different from the national median (P = 0.008), i.e. a stronger difference than in the additive model. Regarding trends over time, the expected annual increase in J&S and Jerusalem regions were longer than the ‘national’ time trend: 28% and 25% respectively (*P* < 0.0001 and *P* = 0.005 for comparison with ‘national’, stronger differences than in the additive model).

In the first quarter of 2022, expected waiting time at the national level was 25 days (95%CI = 21–28), increasing by 26% each year (95%CI = 15-37%). Again, the North region performed better with an expected median waiting time of 16 days (95%CI 13–19) and the Tel-Aviv and J&S regions performed worse than the national level, yielding expected median waiting times of 41 and 39 days respectively.

Regarding trends over time, WTs increased across all regions except for the North and Haifa. Regions with highest increases over time were Centre (+ 36% annual increase) and Jerusalem (+ 39%), with only Jerusalem differing significantly (borderline) from the national median increase (*p* = 0.08).

**Table 3:** Multiplicative piecewise quantile regression model results for log-transformed WT by region presenting relative difference in 2019 and linear trend over time (slope); 2019–2021

Region	Baseline median WT (days) (95% CI)	Relative difference, region vs. national WT	*p*	Slope* (time trend) (95% CI)	*p*	Difference in slope, region vs. national (95% CI)	*P*
National	17.79 (15.56–20.32)			**1.11 (1.06–1.17)**	**< 0.0001**		
South	**14.80** (12.54–17.43)	0.84 (0.72–0.98)	**0.03**	**1.18 (1.10–1.26)**	**< 0.0001**	1.05 (0.98–1.13)	0.15
Haifa	19.23 (15.81–23.34)	1.08 (0.89–1.31)	0.46	1.07 (0.98–1.18)	0.12	0.96 (0.88–1.06)	0.45
Jerusalem	**11.00** (9.08–13.29)	**0.64 (0.53–0.77)**	**< 0.0001**	**1.25 (1.17–1.34)**	**< 0.0001**	**1.12 (1.04–1.22)**	**0.005**
Centre	17.71 (14.81–21.13)	1.00 (0.83–1.20)	0.96	**1.15 (1.06–1.24)**	**0.0004**	1.03 (0.94–1.12)	0.52
North	17.00 (14.25–20.25)	0.96 (0.80–1.15)	0.65	0.98 (0.90–1.06)	0.61	0.88 (0.80–0.97)	**0.008**
Tel Aviv	**22.52 (19.11–26.51)**	**1.25 (1.06–1.48)**	**0.008**	**1.21 (1.13–1.30)**	**< 0.0001**	**1.09 (1.01–1.18)**	**0.038**
J&S	**18.00 (15.65–20.68)**	1.01 (0.87–1.18)	0.89	**1.28 (1.22–1.36)**	**< 0.0001**	**1.15 (1.07–1.24)**	**< 0.0001**

**Bold** indicates significant at the level < 0.007

* indicates expected ratio for median number of days WT per year increase

WT, waiting time; CI, confidence interval, J&S, Judea and Samaria region

**Table 4:** **Multiplicative piecewise quantile regression model results for log-transformed WT by region presenting relative difference in 2022 and linear trend over time (slope); 2022–2023**

Region	Baseline median WT (days) (95% CI)	Relative difference, region vs. national WT	*p*	Slope* (time trend) (95% CI)	*p*	Difference in slope, region vs. national (95% CI)	*P*
National	25.00 (22.12–28.25)			**1.26 (1.15–1.37)**	**< 0.0001**		
South	24.64 (21.20-28.58)	0.98 (0.88–1.11)	0.82	**1.31 (1.19–1.43)**	**< 0.0001**	1.04 (0.94–1.14)	0.44
Haifa	24.07 (20.26–28.56)	0.96 (0.83–1.12)	0.63	**1.08** (0.93–1.25)	0.33	0.86 (0.73–1.01)	0.061
Jerusalem	22.61 (19.62–26.03)	0.91 (0.80–1.03)	0.13	**1.39 (1.26–1.53)**	**< 0.0001**	1.11 (0.99–1.23)	**0.08**
Centre	27.21 (23.40-31.63)	1.09 (0.95–1.24)	0.23	**1.36 (1.20–1.54)**	**< 0.0001**	1.08 (0.96–1.22)	0.22
North	**15.89** (13.40-18.81)	0.65 (0.55–0.76)	**< 0.0001**	1.00 (0.87–1.14)	0.95	0.77 (0.61–0.97)	**0.024**
Tel Aviv	**40.90** (35.54–47.05)	**1.61 (1.43–1.81)**	**< 0.0001**	**1.18 (1.07–1.30)**	**0.001**	0.94 (0.83–1.05)	0.26
J&S	**39.29 (34.86–44.28)**	**1.55 (1.39–1.72)**	**< 0.0001**	1.08 (0.99–1.17)	**0.07**	**0.86 (0.77–0.95)**	**0.004**

#### Sensitivity Analyses of different quantile levels of WT as the outcome (0.5–0.9), based on the Additive Model for 2nd period (2022–2023)

Sensitivity analyses were conducted to look beyond the median values, including 60th, 70th, 80th and 90th percentiles, since WT have a long tail in their distribution. Quantile level of 0.5 is the 50th percentile, i.e. median WT. Detailed results and figures are presented in Appendix B.

### Validation results

Our model demonstrated strong agreement with 2024 data, supporting its robustness. At the national level, the MAE was 1.3 days (Table 5), a relatively low value, accounting for only 4% of the mean of median wait times, which was 30 days - indicating good predictive accuracy. Most regions also showed good agreement between model predictions and actual 2024 WTs (MAE < 20% of the mean of medians WT over three quarters). However, in the Tel-Aviv region, the MAE was quite high (12 days), reflecting poorer agreement. The actual data for Tel Aviv indicated a decline in WT over time in 2024, which the model did not fully capture. Examining predictive accuracy of our model across all regions combined, revealed an MAE of 4.6 days, also relatively low.

**Table 5:** Prediction validation results for 2024 WT data

Region	Quarter 2024	Predicted WT	Actual WT	MAE
National	1	34	34	1.3
National	2	44	44	
National	3	46	50	
Jerusalem	1	38	37	1.0
Jerusalem	2	49	47	
Jerusalem	3	52	52	
South	1	38	35	2.0
South	2	48	48	
South	3	50	53	
Haifa	1	21	22	2.0
Haifa	2	30	30	
Haifa	3	30	35	
Center	1	44	41	5.7
Center	2	55	51	
Center	3	58	48	
North	1	9	14	6.0
North	2	16	21	
North	3	17	25	
Tel-Aviv	1	53	53	12.0
Tel-Aviv	2	63	47	
Tel-Aviv	3	65	45	
J&S	1	42	42	3.3
J&S	2	51	55	
J&S	3	52	58	
**Overall**				4.6

MAE = mean average error

## Discussion

This study was designed to demonstrate a novel statistical application to assess geographical disparities in WT for community-based specialty care in Israel, utilizing dermatology as a case study. Dermatology was chosen as one of the most common specialties, with good national coverage and due to long waiting times for an appointment. The analysis covered all dermatologist practices in the public healthcare system, including both in-person and virtual (synchronous) appointments. The model demonstrated that dermatology WT increased significantly between 2019 and 2023, with growing disparities among regions over time. WTs increased substantially in most regions in the aftermath of the COVID-19 pandemic.

This paper proposes a new statistical application to compare WT for specialists over a period of five years (20 quarters) in the Israeli public health system, using a piecewise quantile regression model. This model, which is not frequently used, was chosen due to its applicability in cases of asymmetric heavy tailed distributions, such as WT. In addition, the piecewise nature of this model enables capturing different trend times in the two periods, which seems to fit our data well. The piecewise quantile regression model enabled statistical quantification of regional disparities and differences in time trends in each examined period, and identification of regions in which WTs are increasing and at what rate, while adjusting for three factors known to impact service supply and demand - COVID lockdowns, seasonality and extreme circumstances such as outbreak of war. Beyond the crude observed median WT seen in the “classical” or basic graphic representation, the model provided expected adjusted median WT and trends over time in each region and period, which are important for resource planning and allocation. This paper may encourage other researchers to apply the quantile regression model and where applicable, the piecewise quantile regression specifically, for analyzing and comparing WTs. Furthermore, while the median was used to model WT, sensitivity analyses were conducted with other percentiles to get a fuller picture of WT trends and differences between regions. Furthermore, the model showed good predictive accuracy for 2024 data.

At the beginning of 2019, the national median WT for an appointment with a specific dermatologist was estimated as two and a half weeks, while in some regions it reached three weeks. By the beginning of 2022, the national median WT for an appointment had climbed to three and a half weeks, in some regions reaching 5–6 weeks. Geographic disparities were demonstrated between regions in those WT. WTs were shown to increase over time at the national level, though not uniformly across regions, with significant increase being observed in all regions except Haifa and the North. In both Tel Aviv and the J&S region, increases in WT were significantly higher than the national level. Indeed, over the five-year period, we see regions maintaining their position, with the same regions with consistently longer waiting time (the central Tel Aviv area and the peripheral J&S region), while the regions with the shortest waiting time for a dermatologist continued to remain the shortest over time (peripheral Northern region), although Jerusalem started with shorter WT and increased significantly more than the national median increase. These results were consistent in both the additive model, utilizing absolute differences of each region and the national level, as well as the multiplicative model, utilizing relative differences. Of note, regions with long WT were not only the peripheral or poorer regions but also the more central and better off areas– WT are indeed affected by many factors, from distribution of physicians, to fluctuating demand. Regional differences were demonstrated not only with median WT but also when looking at 80th and 90th percentiles of WT. In theory, virtual or remote appointments could potentially help to balance WT between regions, eliminating the need to live physically close to a particular practice, however remote (mostly telephone) appointments were included in the current analysis. Asynchronous care i.e. written message to the physician via the app/website, who can respond within several days; or special apps for dermatology care which are provided by some of the HMOs, were not included in the analysis. These options are available and can in some cases provide an alternative to waiting for an appointment and are not dependent on geographical location. A study of a teledermatology app implemented by one of the HMOs found that 30% of patients subsequently booked a clinic appointment for the same problem, indicating that remote services do not eliminate the need for clinic services but can reduce them [18].

Analysis of time trends is crucial to understanding what happens with WT disparities over time. We analyzed the data in two periods, covering the height of the pandemic, and the aftermath (following two years). Given the documented backlogs created during the peaks of COVID-19 activity [19, 20], it is crucial to evaluate the effect of the pandemic on pre-existing disparities in service provision.

Dramatically decreased use and concomitant drops in waiting times for specialists were seen during COVID lockdowns both at the regional level and at the national level, followed by compensatory increases. The model took these drops in WT into consideration by comparing each regional trend to the national trend. Indeed, the data and analysis show a substantial increase in WT during the second period, following the end of the pandemic peak. This suggests that already long wait times rose sharply, with long-term effects– at least in the dermatological specialty.

The overall trend shows WT gradually increasing over time in 2019–2021, with a steeper increase in 2022–2023. Additional follow-up is needed to examine whether this trend will continue and maintain regional disparities in subsequent years, within a healthcare system recovering from the turbulent years of COVID as a health crisis, a period which officially ended in May 2022 [20, 21]. The prediction model for 2024 was fairly accurate for the national level and for most regions, although it overestimated WT for 2024 in Tel Aviv based on the continued increase in previous years. It is possible that the HMOs added resources to somewhat shorten the long WT in Tel Aviv. Another possible explanation is that utilization of newer digital platforms - provided by some HMOs to deal with high demand - is higher among the population in the central, more affluent, Tel-Aviv region, resulting in less demand and shortening WT for the traditional, face-to-face encounter with dermatologists. An example of such a service is a synchronous video consultation with a dermatologist, implemented by one of the largest Israeli HMOs during 2024, in addition to asynchronous tele-dermatology apps utilized in recent years by all HMOs.

Previous research showed extended impact of COVID on waiting times after the end of the pandemic and worsening of disparities [19]. A study in Germany examined WT for appointments in over 200 community-based specialist practices, demonstrating reduced WT during the pandemic due to decreased demand, but with increased regional disparities [22].

As mentioned above, this study is among the first to use quantile regression to compare WTs. Thanh et al. (2013) also compared WT trends in Canada using a similar quantile regression [12], comparing yearly median WT for 3 consecutive years, however they did not further expand the analysis by quarters. In the current study the outcome was the median WT per quarter, allowing a greater resolution. We also analyzed time trends with expected change per year, while controlling for quarter-specific characteristics including COVID lockdowns and seasonality. Since individual patient characteristics were not available, the method assessed geographical differences and examined both time trends within each region, and between regions by comparing each region to the national level. The current study also included sensitivity analyses with greater than median quantile levels.

The use of both additive and multiplicative models provides information on both absolute and relative differences. This is important for providing a more complete picture. It has been suggested that relative inequality measures be used for detecting health inequality and following its magnitude over time, for example to evaluate the effect of interventions to increase equity, while absolute measures can be utilized to take into account disparities in overall population health for different groups, which might assist in quantifying the resources required to implement those interventions [7]. Indeed, there are differences in health outcomes between central and peripheral regions of Israel, as demonstrated by an official publication issued by the Central Bureau of Statistics in 2019 et al. [23] with higher rates of poverty, higher rates of obesity and smoking and poorer perceived health status in the peripheral, compared with the central regions.

The study benefits from the inclusion of all dermatology practices in the public health system, without selection bias. One limitation of the current study is that the model assumes a uniform increase per quarter throughout each of the two periods, i.e. linearity. Second, waiting times are based on actual visits and available appointments in physicians’ scheduling diaries and do not take into account the amount of people who give up on waiting for a public appointment and go private to cut the queue, due to long WTs. On the other hand, true WT is not available for those who ‘walk-in’ - that is turn up without an appointment, however the calculation of WT does take them into consideration by including walk-in visits in the calculation of demand. We assume that these two opposite trends balance each other out. Data on WT for private dermatology appointments were not included since the focus was on the public health system.

## Conclusions and policy implications

Long waiting times can lead to poorer outcomes as well as low patient satisfaction. In dermatology, long WT can lead to patients forgoing treatment and can delay diagnosis of serious conditions where early detection is critical. WT disparities were found within the public health system in peripheral as well as central areas, and a steep spike in waiting times was demonstrated in most regions following the COVID-19 pandemic. Continuous monitoring of WT, at the HMO, regional and national levels, allows analysis of trends and disparities, and the factors affecting them - these are important for efficient allocation of resources, and must be based on solid measurement.

WT is not always measured systematically, and when it is, there is no consensus on the best way to model the data for optimal use. This novel utilization of piecewise quantile regression modelling, used on systematic longitudinal WT measurement, both at the HMO, regional and national levels, demonstrated significant geographic disparities while taking into account seasonality, the effect of a health crisis like COVID-19 and a period of war, and was able to predict WT within a reasonable range. Advanced statistical modelling can highlight changing trends and disparities and contribute to resource planning to help improve system efficiency. Indeed the ratio of dermatologists relative to population size in Israel is lower than other specialties, and did not increase over the years [24].

The health system and HMOs need accurate information on where patients are waiting the longest and in which specialties, to make efforts to synchronize supply and demand for appointments, and increase availability of services where necessary. Future research is being conducted examining which factors might explain longer WT in certain areas, for example looking at number of physician-hours per population and may also examine differences on a smaller geographical level. Since regions with the longest WT are not always the most peripheral or those of low socioeconomic background, the effect of patient behaviour and preferences should be considered, for example whether high demand for dermatologists– and specialists in other fields - may be related to the ability to directly schedule an appointment in specialties which do not require a general practitioner (GP) referral. In some cases, for minor complaints, it may be more appropriate for patients to consult with their GP, and for the GP to refer to a specialist if the complaint warrants further investigation; the GP acting as gatekeeper and reducing the burden on specialists. Furthermore, the HMOs have developed digital apps including for dermatology in an attempt to reduce the burden on physicians. These could be more widely publicized, as well as considering new technological (AI) tools to help direct patients to the most appropriate service for their complaint, which may not always be a face-to-face physician visit.

## Electronic supplementary material

Below is the link to the electronic supplementary material.

Supplementary Material 1

## Data Availability

The data underlying this article can be accessed at the following link which provides national data by specialty (including Dermatology, quarter (2019-Q1/2024), district and locality. https://gis.health.gov.il/waitingtime/.

## Abbreviations

CI: Confidence interval
HMO: Health Maintenance Organization
WT: Waiting time(s)
J&S: Judea and Samaria
GP: General practitioner
