# Supplementary material for: Measuring geographical disparities in waiting times for community-based specialist care - a novel statistical application
Source: Isr J Health Policy Res. 2025 Jul 14;14:43. doi: 10.1186/s13584-025-00702-7 (PMC12257811; doi:10.1186/s13584-025-00702-7)
Supplement: Supplementary file 1 — Supplementary Material 1 [file 13584_2025_702_MOESM1_ESM.docx]

**Appendix A – Piecewise linear regression**

Consider the following plot (1) of the compressive strength (y) of n = 18 batches of concrete against the proportion of water (x) mixed in with the cement. The solid line is a linear regression:

Y=$\alpha+\beta_{1}X+\varepsilon$


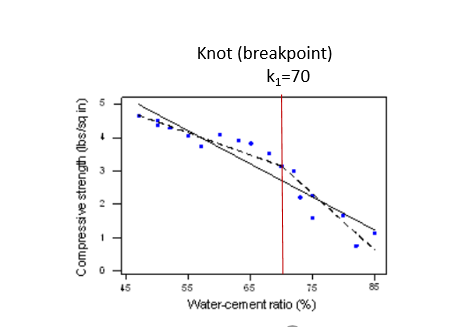


The dashed line is a Piecewise regression as follows:

Y=$\alpha+\beta_{1}X+\beta_{2}\left( X-k_{1} \right)*I_{\left[ x>k1 \right]}+\varepsilon$

Where the knot (piecewise divider), $k_{1}$ equals 70. This model enables the following different intercept and slope for each of the pieces (periods):

$$Intercept=\left\{ \begin{matrix} \alpha; & X\leq70 \\ \alpha-70*\beta_{2} ; & X>70 \end{matrix} \right\}$$

$$slope=\left\{ \begin{matrix} \beta_{1} ; & X\leq70 \\ \beta_{1}+\beta_{2} ; & X>70 \end{matrix} \right\}$$

As can be seen, the piecewise model (dashed lines) fits the data better.

**Piecewise quantile regression**


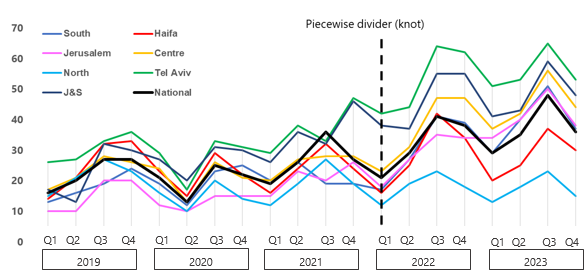


Suppose we had only waiting times (WT) data of the national level.

The piecewise quantile regression can be written as follows:

Median(WT)=$\alpha+\beta_{1}*Q+\beta_{2}*\left( Q-13 \right)*I_{\left[ Q>13 \right]}+\varepsilon$ ;

Where Q=1,2, …., 20 , and Q=13 is the knot for the piecewise regression - i.e. the 1^st^ quartile of 2022

2019-2021 Median(WT)=$\alpha+ \beta_{1}*Q+\varepsilon$

2022-2023 Median(WT)=($\alpha-13*\beta_{2})+ {(\beta}_{1}+\beta_{2})*Q+\varepsilon$

To calculate median WT at first quarter of 2019, Q equals 1. To calculate median WT at first quarter of 2022, Q equals 13.

To calculate trend per year, in the first period: 4*$\beta_{1}$ and in the second period: 4*(${(\beta}_{1}+\beta_{2})$.

Now, suppose we had WT data of one region, Jerusalem, and WT data on the national level as well, where the reference category is national, as follows:

$$Region=\left\{ \begin{matrix} 0 ; & National \\ 1 ; & Jerusalem \end{matrix} \right\}$$

The quarter is Q=1,2, …., 20, where Q=13 is the knot for the piecewise regression.

The piecewise quantile regression can be written as follows:

Median(WT)=$\alpha+{\beta_{1}*Region+\beta}_{2}*Q+\beta_{3}*Region{*Q+\beta}_{4}*\left( Q-13 \right)*I_{\left[ Q>13 \right]}$

${+\beta}_{5}*Region*\left( Q-13 \right)*I_{\left[ Q>13 \right]}+\varepsilon$ ;

For National:

2019-2021 Median(WT)=$\alpha+ \beta_{2}*Q+\varepsilon$

2022-2023 Median(WT)=($\alpha-13*\beta_{4})+ {(\beta}_{2}+\beta_{4})*Q+\varepsilon$

For Jerusalem region:

2019-2021 Median(WT)=$\alpha+\beta_{1}+ {(\beta}_{2}+\beta_{3})*Q+\varepsilon$

2022-2023 Median(WT)=$\alpha+\beta_{1}-13*{(\beta}_{4}{+\beta}_{5})+ {(\beta}_{2}+\beta_{3}+\beta_{4}{+\beta}_{5})*Q+\varepsilon$

To calculate median WT at first quarter of 2019, Q equals 1. To calculate median WT at first quarter of 2022, Q equals 13.

To calculate trend per year:

On national level in the first period: 4*$\beta_{2}$ and in the second period: 4*${(\beta}_{2}+\beta_{4})$.

In Jerusalem region, in the first period: 4*${(\beta}_{2}+\beta_{3})$ and in the second period:

$4*{(\beta}_{2}+\beta_{3}+\beta_{4}{+\beta}_{5})$.

This can be easily generalized to seven regions and national level as the reference category.

All models were adjusted for quarters in which COVID-19 lockdowns occurred (Q2 2020/Q1 2021) or outbreak of war (Q4 2023).

**Appendix B – Sensitivity Analyses for different quantile levels of WT as the outcome (0.5-0.9), based on the Additive Model for 2^nd^ period (2022-2023)**

Sensitivity analyses were conducted to look beyond the median values, including 60^th^, 70^th^, 80^th^ and 90^th^ percentiles, since WT have a long tail in their distribution. Quantile level of 0.5 is the 50^th^ percentile, i.e. median WT.

Figure 2 presents the estimated baseline national WT at the 1^st^ quarter of 2022. The blue shade indicates the 95% Confidence Intervals (CI). At the 50^th^ percentile, baseline median WT is 24.5 days and it increases as the quantile level increases (69 days for the 90^th^ percentile) (ie. 50% of appointments were within 24.5 days and 90% of appointments were with 69 days’ wait).

Figure 3 presents the difference between the baseline WT in the North region and the national baseline WT, for different quantile levels. The graph shows that the upper CI of the difference between the North and national WT is below zero across the board from 50^th^-90^th^ percentile. This means that the difference in baseline WT between North and the national level is usually negative, and therefore WT in the North at the beginning of 2022 was shorter than National, even at the extremes of 80^th^ and 90^th^ percentiles of WT.

Figure 2: Estimated baseline National WT 2^nd^ period (Q1 2022) and 95% Confidence Limits

by quantile level


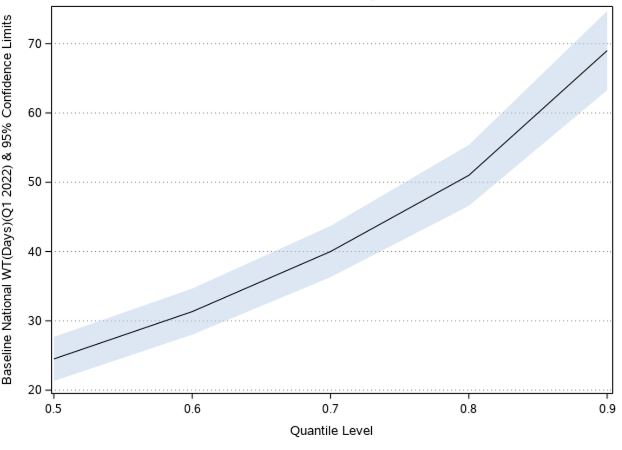


Figure 3: Difference between North region and national WT (days) at 1^st^ quartile of 2022


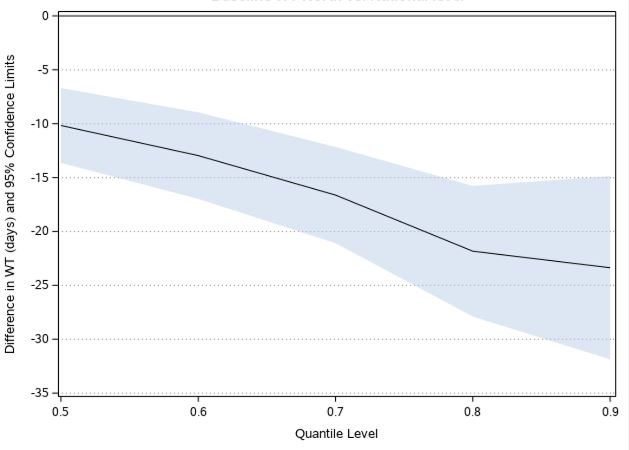


Figure 4, presents the difference between the a) baseline WT in the Tel-Aviv region and the national level, and b) in J&S region and the national level, for different quantile levels. These graphs show that the lower CI of the difference is above zero, indicating that for both regions the difference in baseline WT from the national level is usually positive, demonstrating longer WT at the beginning of 2022 than National, even at the extremes of 80^th^ and 90^th^ percentiles of WT.

Figure 4: Difference in baseline WT (Q1 2022) between Tel-Aviv region and 'National' (a), J&S and 'National' (b)


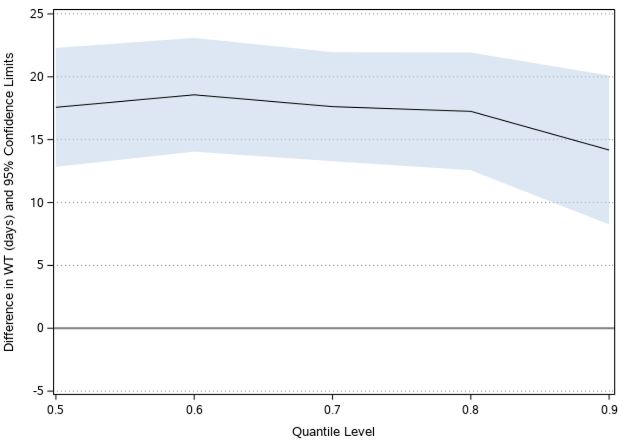

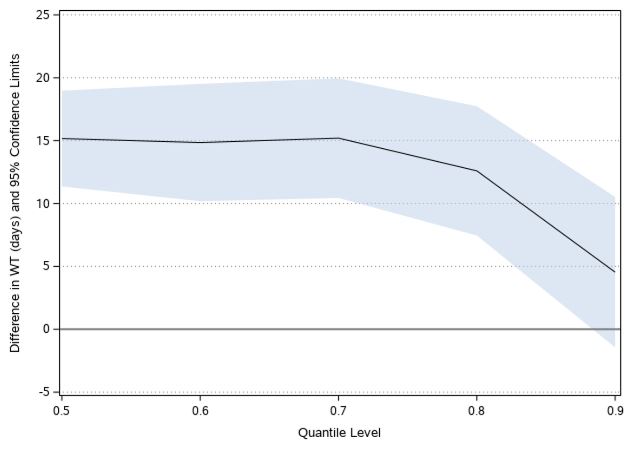


Figure 5 presents the time trend for WT at the national level for different quantile levels. The graph shows that WT increases by 8.5 days per one year increase for the median (50^th^ percentile). This increase in WT per year decreases at the extremes of 80^th^ and 90^th^ percentiles of WT (e.g. only 5 days increase per year at the 90^th^ percentile).

Figure 5: Estimated national WT slope (annual increase in WT days) by quantile Level


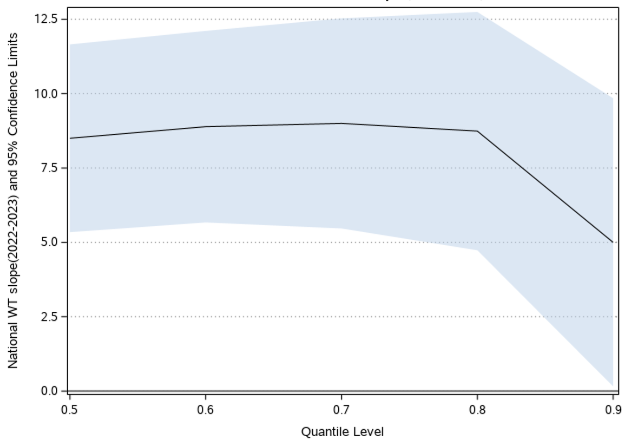


Figure 6 presents the difference between the time trend of WT in the Jerusalem region and the national level for different quantile levels. The graph shows that zero is inside the 95% CI for all quantiles levels. In other words, the change in WT over time in the Jerusalem region is not statistically different from the change at the national level.

**References:**

1. Penn. Stat 501 Regression methods. https://online.stat.psu.edu/stat501/lesson/8/8.8. Penn state, Eberly College of Science.
